# Increased oxidized low‐density lipoprotein in mice exposed to a high‐fat diet impaired spermatogenesis by inhibiting testosterone synthesis via the Klk1bs/Eid3 pathway

**DOI:** 10.1002/ctm2.1603

**Published:** 2024-03-03

**Authors:** Chenfeng Yuan, Hanhui Hong, Nan Wang, Tong Chen, Maosheng Cao, Yun Zhao, Caomeihui Shen, Xue Chen, Yuxin Luo, Boqi Zhang, Xu Zhou, Chunjin Li

**Affiliations:** ^1^ College of Animal Sciences Jilin University Changchun P. R. China; ^2^ College of Biological Sciences and Technology Beijing Forestry University Beijing P. R. China

Dear Editor,

The adverse impact of obesity on male reproduction has been widely recognized, but there is no consensus regarding the underlying mechanisms. In this study, a comprehensive transcriptome analysis of different cell subpopulations from the testes of normal diet (ND) and high‐fat diet (HFD) mice was performed using single‐cell RNA sequencing to explore the possible mechanisms by which HFD affects male fertility reproduction (Figure 1A).

**FIGURE 1:**
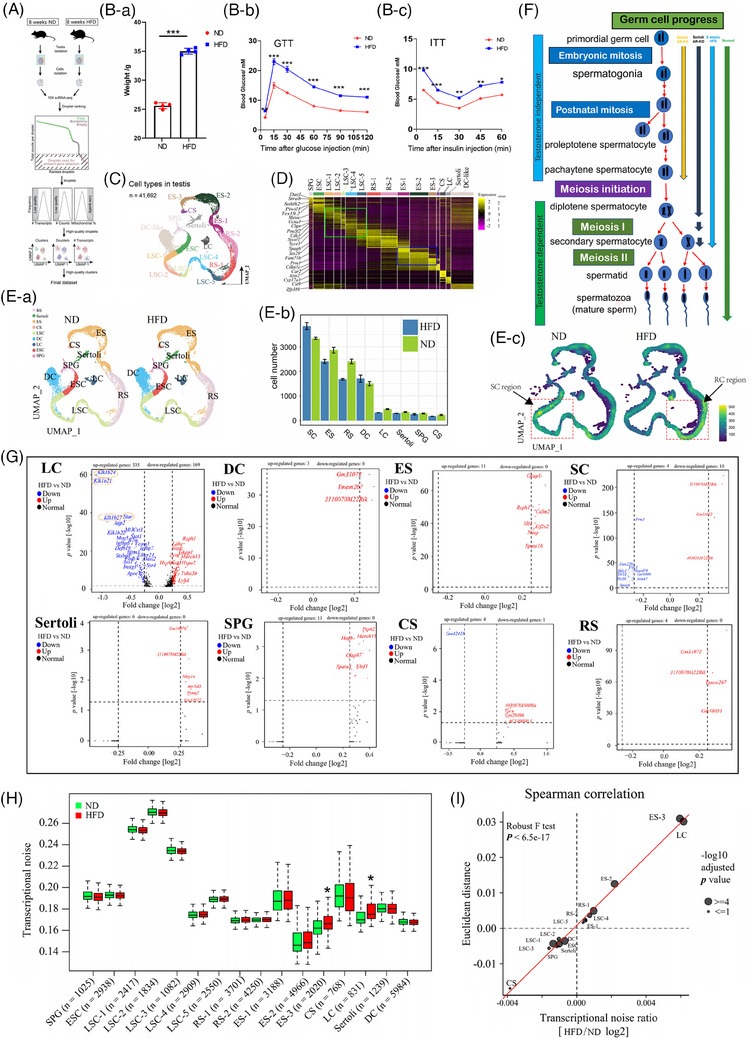
Single‐cell RNA (scRNA) sequencing of testis cells in the ND and HFD groups. (A) Outline of the workflow. (B‐a) Weight of mice in the HFD and ND groups. (B‐b) Blood glucose during the GTT (*n* = 5 per group). (B‐c) Blood glucose during the ITT (*n* = 5 per group). (C) UMAPs of types of testis cells. (D) The top 10 marker genes per cell cluster in the scaled expression matrix. The column and row labels represent the cell clusters identified according to the average log‐transform fold change value, spermatogonia (SPG), early spermatocytes (ESC), late spermatocytes (LSC‐1, LSC‐2, LSC‐3, LSC‐4 and LSC‐5), round spermatocytes (RS‐1 and RS‐2), elongated spermatids (ES‐1, ES‐2 and ES‐3), condensed spermatozoa (CS), Leydig cells (LC), Sertoli cells and dendritic (DC) cells. (E‐a) UMAPs of types of testis cells in the ND and HFD groups. (E‐b) Numbers of cell types in the ND and HFD groups. (E‐c) Distribution of cell density for each type of cell. (F) Effects of different ways of blocking the testosterone signaling pathway on spermatogenesis. (G) Volcanic maps of the differential genes in each cell type. (H) Boxplot delineating transcriptional noise in the obese and control groups according to cell type with the number of cells indicated. (I) Scatterplot depicting the log2 ratio of transcriptional noise calculated for the obese and control samples. GTT, glucose tolerance test; HFD, high‐fat diet; ITT, insulin tolerance test; ND, normal diet; UMAP, Uniform Manifold Approximation and Projection.

After eight weeks of feeding, mice in the HFD group showed significant weight gain, glucose tolerance and insulin resistance (Figure 1B). According to the experimental workflow (Figure 1A), high‐quality transcriptomic data were obtained from 41,692 cells. By applying data filtering and uniform manifold approximation projection (UMAP) analysis, these cells were classified into 16 clusters (Figure 1C), and based on previous studies and specific gene expression, these cells were classified into nine types (Figure 1D and Figure S1A). We also found that HFD mice had more spermatocytes and fewer sperm (Figure 1E), which had characteristics of spermatogenesis arrest. It is well established that HFD can lead to decreased testosterone (Figure S1B) and that the loss of testosterone signalling in the testes can lead to spermatogenesis arrest (Figure 1F).^1^ To learn more about how an HFD damages testicular cells, we analyzed transcriptional profiles in individual cells (Figure 1G), and we found a total of 594 differentially regulated genes, 563 of which are occupied by Leydig cells. A significant increase in transcriptional noise in Leydig cells was also observed (Figure 1H), which was supported by the results of Spearman correlation analysis in all cells (Figure 1I and Figure S1C). Therefore, we believe that the damage of HFD to male fertility was concentrated in Leydig cells.

We focused on the Leydig cells to study the specific mechanisms of HFD damage to them. We found that some important mitochondrial DNA and steroid synthesis‐related genes were significantly downregulated in the HFD group (Figure 2A). We also found some differential genes (Klk1b21/24/27 and Eid3) that have been rarely reported in reproduction but might have important functions therein (Figure 2A and Figure S1D).^2^, ^3^, ^4^ We constructed a Monocle3 heatmap to broaden the scope of the transcriptomic landscape (Figure 2B), and we divided Leydig cells into three subclusters with special markers (Figure 2C). Klk1b21/24/27 was significantly downregulated in HFD Leydig cells in all study periods (Figure 2D), and the proportion of mature Leydig cells was slightly reduced in HFD mice (Figure S1E). However, the specific substances through which the HFD impairs Leydig cells are not known. Through enrichment analysis of differential genes, we found that the cholesterol homeostasis pathway was significantly downregulated in HFD Leydig cells (Figure 2E). As previously described,^5^ we examined low‐density lipoprotein (LDL) and oxidized LDL (ox‐LDL) levels in serum and testes, and we found more LDL and oxidized LDL in HFD mice (Figure S1F and Figure 2F). Next, we added LDL or ox‐LDL to the culture medium of Leydig cells and found that LDL significantly promoted the expression of mitochondrial DNA, steroid synthesis‐related genes, and Klk1b21/24/27 in Leydig cells, and also decreased the expression of Eid3 (Figure S1G,H), promoted the expression of steroid synthesis‐related gene proteins (Figure S1I), and suppressed testosterone synthesis in Leydig cells (Figure S1J). However, ox‐LDL significantly decreased the expression of mitochondrial DNA, steroid synthesis‐related genes, and Klk1b21/24/27 in Leydig cells, and also promoted the expression of Eid3 (Figure 2G,H). The primer sequences were listed in Tables S5. In further assays, we found that ox‐LDL reduced the mitochondrial membrane potential of Leydig cells (Figure 2I), inhibited the expression of steroid synthesis‐related gene proteins (Figure 2J), and suppressed testosterone synthesis in Leydig cells (Figure 2K). These results suggested ox‐LDL to be an important substance mediating the impairment of Leydig cell function by an HFD.

**FIGURE 2:**
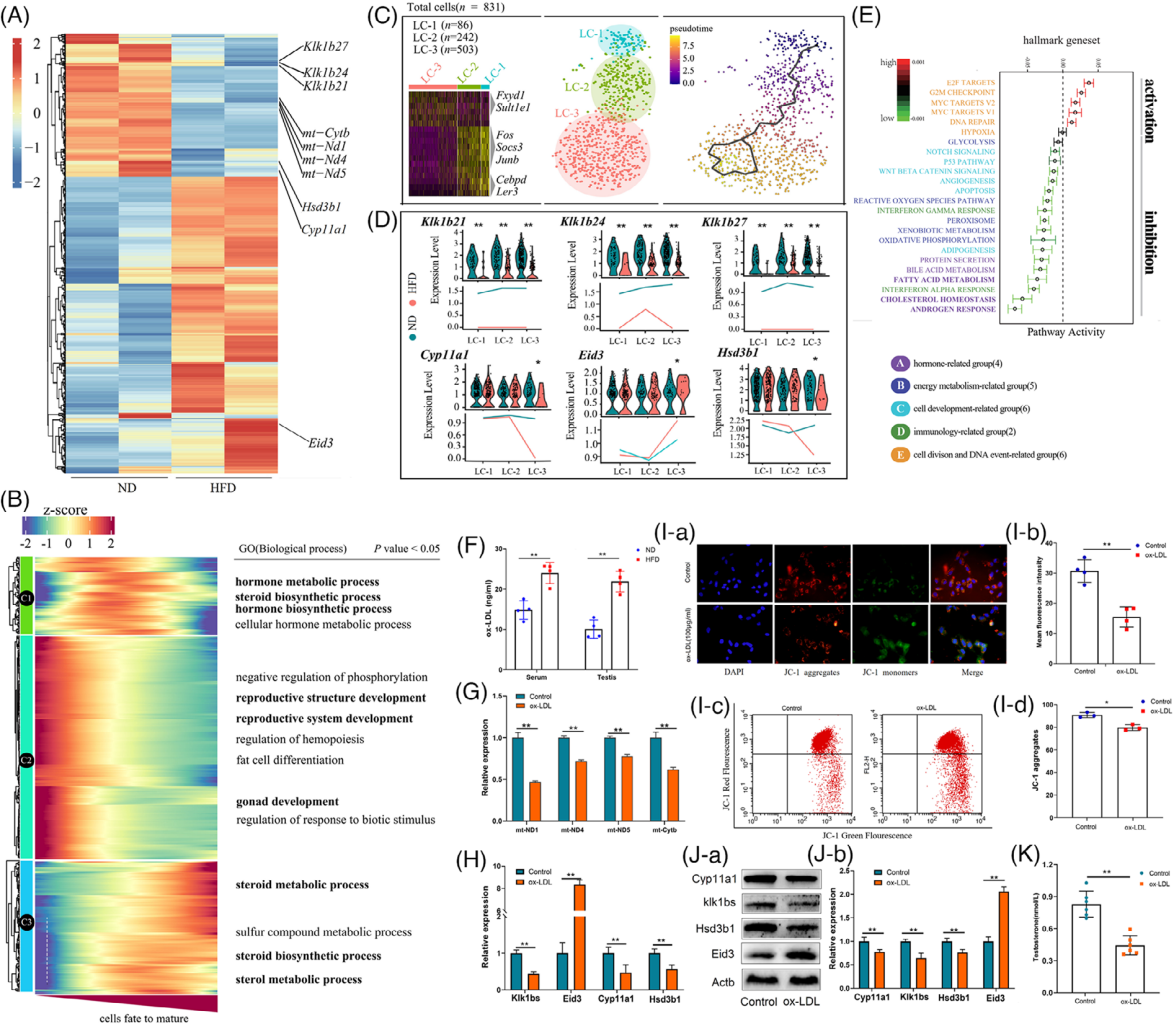
Disruption of Leydig cell function by oxidized low‐density lipoprotein. (A) Heat maps of differential genes in Leydig cells in the HFD and ND groups. (B) The pseudo‐time heatmap classified into three clusters by the hierarchical method. GO analysis was performed for each cluster. (C) Heatmap displaying significant marker genes in each cluster. UMAP of Leydig cells classified into three clusters (i.e., LC‐1, Cebpd+ cluster; LC‐2, Fos+ cluster; LC‐3 and Sult1e1+ cluster). Development of Leydig cells predicted by RNA velocity. (D) Violin plots of Klk1b21, Klk1b24, Klk1b27, Cyp11a1, Eid3 and Hsd3b1 in the HFD and ND groups. (E) Boxplot profiling pathway activity in Leydig cells in the obese and control groups. (F) Serum and testicular ox‐LDL levels were measured by enzyme‐linked immunosorbent assay (*n* = 4 per group). (G) mRNA expression of mt‐ND1, mt‐ND4, mt‐ND5 and mt‐Cytb was examined by RT‐qPCR (*n* = 3 per group). (H) mRNA expression of Klk1bs, Eid3, Cyp11a1 and Hsd3b1 was examined by RT‐qPCR (*n* = 3 per group). (I‐a, I‐b) MMP in Leydig cells was assessed using JC‐1 staining (*n* = 4 per group). (I‐c, I‐d) MMP was assayed by flow cytometry (*n* = 3 per group). (J‐a, J‐b) Protein expression of Klk1bs, Eid3, Cyp11a1 and Hsd3b1 was detected by Western blotting (*n* = 3 per group). (K) Testosterone levels in cell culture medium were measured by enzyme‐linked immunosorbent assay (*n* = 6 per group). GO, Gene Ontology; HFD, high‐fat diet; ND, normal diet; UMAP, Uniform Manifold Approximation and Projection; HFD, high‐fat diet; MMP, mitochondrial membrane potential; ND, normal diet; ox‐LDL, oxidized low‐density lipoprotein; RT‐qPCR, real‐time quantitative polymerase chain reaction.

To investigate the functions of Klk1bs and Eid3 in Leydig cells, we constructed a Klk1bs knockdown vector (Tables S1 ,Tables S4 and Figure S1G) and an Eid3 overexpression adenovirus vector (Tables S2 and Figure S1H), respectively. In vitro, we found that Klk1bs knockdown or Eid3 overexpression in Leydig cells significantly suppressed the expression of steroid synthesis‐related genes (Figure 3A,B,J,K), which in turn inhibited testosterone synthesis (Figure 3C,L). In vivo, the knockdown of Klk1bs or overexpression of Eid in Leydig cells by testicular injection resulted in lower expression of steroid‐synthesizing genes in the testis (Figure 3D,E,M,N), which led to lower levels of serum testosterone (Figure 3F,O), lower sperm count in the epididymis (Figure 3G,P), and severe disruption of seminiferous tubules (Figure 3H,Q). Immunohistochemical staining showed a significant decrease in the number of sperm and the total number of germ cells in the testis (Figure 3I,R), suggesting that Klk1bs and Eid3 have a significant effect on spermatogenesis by regulating testosterone synthesis.

**FIGURE 3:**
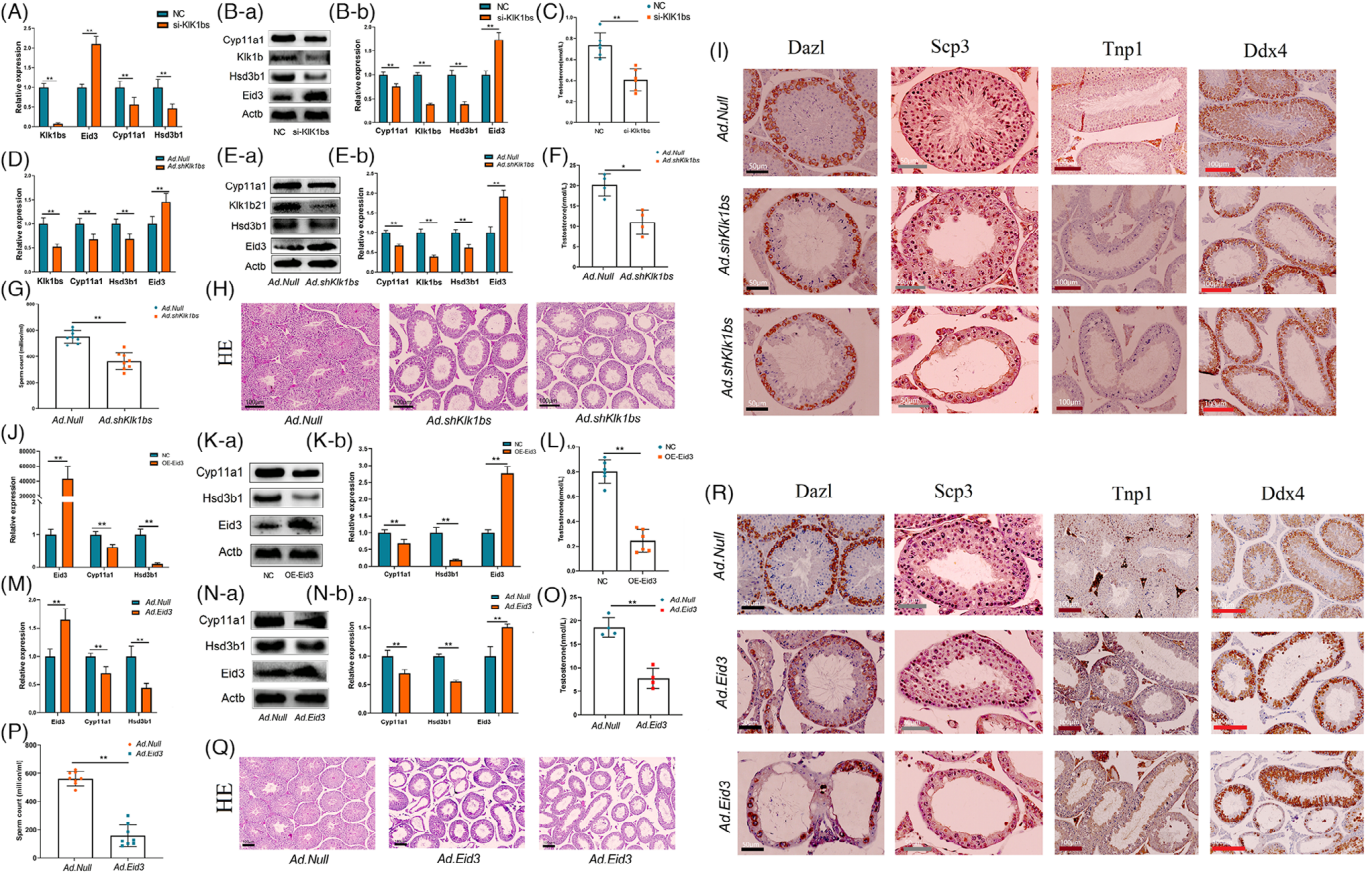
Klk1b21/24//27 and Eid3 are important genes regulating testosterone synthesis in Leydig cells. (A) mRNA expression of Klk1bs, Eid3, Cyp11a1 and Hsd3b1 was examined by RT‐qPCR (*n* = 3 per group). (B) Protein expression of Klk1bs, Eid3, Cyp11a1 and Hsd3b1 was detected by Western blotting (*n* = 3 per group). (C) Testosterone levels in cell culture medium were measured by enzyme‐linked immunosorbent assay (*n* = 6 per group). (D) mRNA expression of Klk1bs, Eid3, Cyp11a1 and Hsd3b1 was examined by RT‐qPCR (*n* = 3 per group). (E) Protein expression of Klk1bs, Eid3, Cyp11a1 and Hsd3b1 was detected by Western blotting (*n* = 3 per group). (F) Serum testosterone levels were detected by enzyme‐linked immunosorbent assay (*n* = 4 per group). (G) Sperm from each cauda epididymis in Ad.Null and Ad.shKlk1bs mice were counted in a hemocytometer under a light microscope (*n* = 8 for each group). (H) Hematoxylin and eosin staining of histological sections of testes in Ad.Null and Ad.shKlk1bs mice (*n* = 3 per group; scale bar, 200 μm). (I) Immunohistochemical staining of histological sections of testes in Ad.Null and Ad.shKlk1bs mice (*n* = 3 per group), Dazl, spermatogonium; Scp3, spermatocyte; Tnp1, sperm cell; Ddx4, germ cell. (J) mRNA expression of Eid3, Cyp11a1 and Hsd3b1 was examined by RT‐qPCR (*n* = 3 per group). (K) Protein expression of Eid3, Cyp11a1 and Hsd3b1 was detected by Western blotting (*n* = 3 per group). (L) Testosterone levels in cell culture medium were measured by enzyme‐linked immunosorbent assay (*n* = 6 per group). (M) mRNA expression of Eid3, Cyp11a1 and Hsd3b1 was examined by RT‐qPCR (*n* = 3 per group). (N) Protein expression of Eid3, Cyp11a1 and Hsd3b1 was detected by Western blotting (*n* = 3 per group). (O) Serum testosterone levels were detected by enzyme‐linked immunosorbent assay (*n* = 4 per group). (P) Sperm from each cauda epididymis in Ad.Null and Ad.Eid3 mice were counted in a hemocytometer under a light microscope (*n* = 8 for each group). (Q) Hematoxylin and eosin staining of histological sections of testes in Ad.Null and Ad.Eid3 mice (*n* = 3 per group; scale bar, 200 μm). (R) Immunohistochemical staining of histological sections of testes in Ad.Null and Ad.Eid3 mice (*n* = 3 per group), Dazl, spermatogonium; Scp3, spermatocyte; Tnp1, sperm cell; Ddx4, germ cell.

In an attempt to rescue the reproductive damage due to HFD, we constructed a Klk1b21 overexpression vector (Tables S3 and Figure S1I). In vitro, we first demonstrated that Klk1b21 overexpression in Leydig cells promoted the expression of steroid synthesis‐related genes (Figure 4A,B) and testosterone synthesis (Figure 4C). In vivo, we found that increasing Klk1b21 expression in the testes of HFD mice rescued the expression of steroid synthesis‐related genes (Figure 4D,E), increased serum testosterone levels (Figure 4F), restored the disruption of the structure of seminiferous tubules brought about by the HFD (Figure 4G), and increased the sperm count in the epididymis of HFD mice (Figure 4H). Immunohistochemical staining showed that increasing Klk1b21 expression in the testes of HFD mice significantly increased the sperm count and the total number of germ cells in the testes of HFD mice (Figure 4I).

**FIGURE 4:**
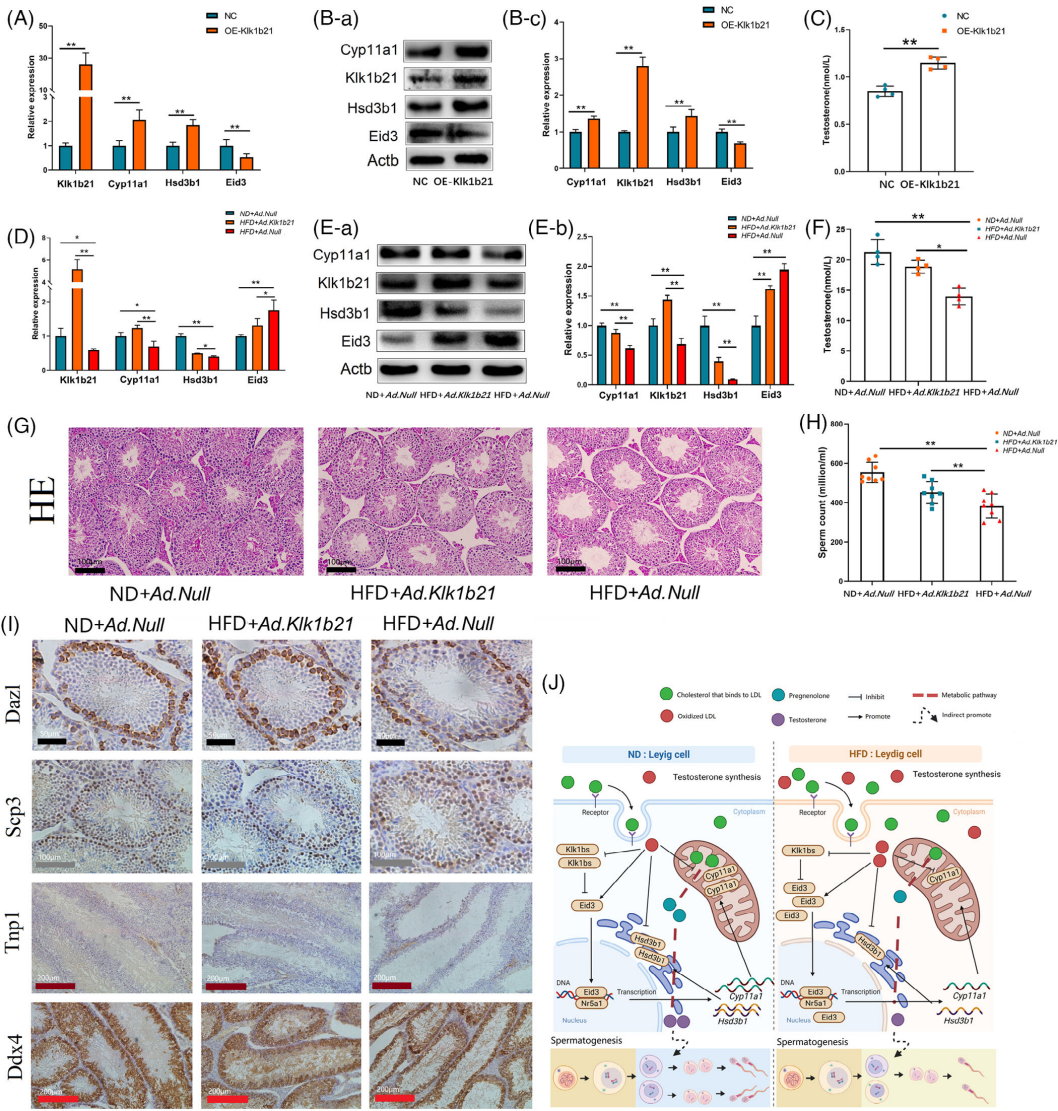
Supplementation with Klk1b21 can reverse reproductive damage caused by HFD. (A) mRNA expression of Klk1b21, Eid3, Cyp11a1 and Hsd3b1 was examined by RT‐qPCR (*n* = 3 per group). (B) Protein expression of Klk1b21, Eid3, Cyp11a1 and Hsd3b1 was detected by Western blotting (*n* = 3 per group). (C) Testosterone levels in cell culture medium were measured by enzyme‐linked immunosorbent assay (*n* = 4 per group). (D) mRNA expression of Klk1b21, Eid3, Cyp11a1 and Hsd3b1 was examined by RT‐qPCR (*n* = 3 per group). (E) Protein expression of Klk1bs, Eid3, Cyp11a1 and Hsd3b1 was detected by Western blotting (*n* = 3 per group). (F) Serum testosterone levels were detected by enzyme‐linked immunosorbent assay (*n* = 4 per group). (G) Hematoxylin and eosin staining of histological sections of testes in ND + Ad.Null, HFD + Ad.Klk1b21 and HFD + Ad.Null mice (*n* = 3 per group; scale bar, 200 μm). (H) Sperm from each cauda epididymis in ND + Ad.Null, HFD + Ad.Klk1b21 and HFD + Ad.Null mice were counted in a hemocytometer under a light microscope (*n* = 8 in each group). (I) Immunohistochemical staining of histological sections of testes in ND + Ad.Null, HFD + Ad.Klk1b21 and HFD + Ad.Null mice (*n* = 3 per group). Dazl, spermatogonium; Scp3, spermatocyte; Tnp1, sperm cell; Ddx4, germ cell. (J) A high‐fat diet increased the ox‐LDL level in Leydig cells, which resulted in a decreased ability to synthesize testosterone in Leydig cells, and the decreased testosterone concentration in the testis caused spermatogenic arrest.

Overall, we found that the HFD induced the accumulation of ox‐LDL in Leydig cells, which impaired the testosterone synthesis ability of Leydig cells, leading to spermatogenesis arrest in the testes. In the rescue experiment, we found that increasing Klk1b21 expression partially rescued the reproductive damage in mice. This study provides a theoretical basis for potential clinical treatment of infertility in obese men (Figure 4J).

## AUTHOR CONTRIBUTIONS

***Conceptualization***: Chunjin Li, Xu Zhou, Chenfeng Yuan; ***Methodology***: Nan Wang, Tong Chen, Hanhui Hong; ***Investigation***: Maosheng Cao, Yun Zhao, Caomeihui Shen; ***Visualization***: Xue Chen, Yuxin Luo, Yun Zhao, Boqi Zhang; ***Writing—original draft***: Chenfeng Yuan, Hanhui Hong; ***Writing—review & editing***: Chunjin Li, Xu Zhou.

## CONFLICT OF INTEREST STATEMENT

The authors declare no conflict of interest.

## FUNDING INFORMATION

National Natural Science Foundation of China, 32172726 and 32272872. Key Research and Development Program of Jilin Province, 20210202103NC, 20210202048NC and 20190301008NY. Outstanding Young Talents and Technology Innovation Project of Jilin Province, 20220508094RC. Key Research and Development Program of Changchun City, 21ZGN05.

## ETHICS STATEMENT

The animal study was reviewed and approved by the Animal Ethics Association of Jilin University (JLU: SY202002080).

## Supporting information

Supporting Information

Figure S1: High‐fat diet impaired spermatogenesis by inhibiting testosterone synthesis.

Supporting Information

## Data Availability

The data presented in the study are deposited in the NCBI repository, accession number GSE254812.
