# Supplementary material for: Increased oxidized low‐density lipoprotein in mice exposed to a high‐fat diet impaired spermatogenesis by inhibiting testosterone synthesis via the Klk1bs/Eid3 pathway
Source: Clin Transl Med. 2024 Mar 3;14(3):e1603. doi: 10.1002/ctm2.1603 (PMC10909978; doi:10.1002/ctm2.1603)
Supplement: Supplementary file 1 — Supporting Information [file CTM2-14-e1603-s003.docx]

**1 Materials and methods**

1.1 Animals

All experimental procedures were approved by the Animal Protection and Use Committee (SY202002080

). Ten 21-day-old male C57BL/6 mice were purchased from the Model Animal Research Center of Jilin University and used to establish the HFD model and for other in vivo experiments. The mice were kept in a specific pathogen-free environment on a 12-hour diurnal cycle with free access to food and water and were randomized to receive an ND or HFD for 8 weeks to establish mouse models of ND and HFD, after which they were used for the experiments.

1.2 scRNA-seq library preparation and sequencing

Cell populations were isolated from the testes of mice in the ND and HFD groups. In brief, the albuginea was removed, and the tissue was incubated in dissociation buffer containing collagenase IV (Sigma-Aldrich, St Louis, MO, USA; 1 mg/mL), dispase II (10 mg/mL), and DNase I (DN25, Sigma; 50,000 U/mL) for 30 min at 37°C. Enzymatic digestion was quenched with Dulbecco’s Modified Eagle’s Medium (Gibco, Carlsbad, CA, USA) supplemented with 10% fetal calf serum. Before loading, the cell viability of each sample was evaluated by staining an aliquot with Trypan Blue, and the viability was typically determined to be greater than 80%. The cells were subsequently loaded into each channel of a Chromium Single Cell B Chip (10x Genomics, Pleasanton, CA, USA), and approximately 13,000 cells were recovered from each channel. A Chromium Single Cell 3ʹ Library and Gel Bead Kit using v3 Chemistry (10X Genomics) was used for single-cell barcoding, synthesis of complementary DNA, and library preparation following the manufacturer’s protocol (Chromium Single Cell 3ʹ Reagent Kits v3 User Guide, Revision B). The libraries were sequenced on an Illumina NovaSeq 6000 system using paired-end sequencing runs.

1.3 Processing of single-cell sequencing data

Paired-end reads were first separated by the specific cell barcode information in read 2. The corresponding read 1 was aligned based on the UMI information. The TSO sequence, poly(A) tail sequence, reads with adaptor contaminants, and low-quality bases (N >10%) were removed to obtain clean reads. Next, the clean reads were aligned to the GRCm38 transcriptome (UCSC) by the Cellranger (v3.1.0) 10X pipeline (https://support.10xgenomics.com/single-cell-gene-expression/software/pipelines/latest) and a count matrix was generated. Cell-specific barcodes were used to group the uniquely mapped reads71. Duplicated transcripts were removed based on UMI information. Finally, we counted distinct UMIs as the transcript copy number.

1.4 Identification of differentially expressed genes

We used the Seurat (https://satijalab.org/seurat/) FindAllMarkers function based on log-normalized expression values to identify differentially expressed genes between two groups of cells using the Wilcoxon rank sum test. The Wilcoxon test was conducted on each individual gene to return the p-value; a positive average log-transform fold change represents upregulation in the corresponding clusters. Filtered genes with a p-value adjusted to 0.05 were selected. GO analysis was performed using clusterProfiler74. GO terms with a p-value <0.05 were considered statistically significant.

1.5 Lineage reconstruction via RNA velocity, PAGA, and Monocle3

RNA velocities (http://velocyto.org/) were computed via velocyto under default parameters to determine the start and end points of trajectories. The trajectories were presented using the PAGA (https://github.com/theislab/paga) and Monocle3 (<http://cole-trapnell-lab.github.io/monocle-release/monocle3/>) methods.

1.6 Gene set variation analysis

We applied gene set variation analysis (GSVA 1.38.2)78 to eight pathways from the mouse misgdbr v7.2.1 of the GO database. The normalized cluster data (LSC-3 and LSC-4) were used to calculate the means of all cells among the obese and control groups. We then visualized it with the heatmap.

1.7 Isolation and culture of primary Leydig cells

Leydig cells were isolated using a previously described method(1). Briefly, after opening the abdominal cavity and taking out the testes, the mice testes were opened with eyebrow tweezers, gently dispersed, and placed in a 6-cm cell culture dish. Next, 5 mL of digestive solution (0.05% type I collagenase + 1% bovine serum albumin + 98.95 phosphate-buffered saline [PBS]) were added. The specimens were then placed in a cell incubator equipped with a shaker and digested at 100 rpm for 15 min. After digestion, 5 mL of cell culture medium (DMEMF-12 + 10% fetal bovine serum) and 20 mL PBS were added to terminate digestion. Next, the samples were centrifuged at 300 g for 5 minutes. The supernatant was then discarded, and 12 mL of cell culture medium were added to re-suspend the cells. The cells were cultured on cell culture dishes with a diameter of 10 cm, which were then placed in a cell incubator at 37°C in 5% carbon dioxide and full humidity. After 24 h of cell culture, the cells were cleaned in PBS, placed in fresh medium, and cultured for a further 48 h. Finally, the Leydig cells were digested and inoculated on 12-well (4 × 10^4^ cells/well) or 6-well (4 × 10^5^ cells/well) cell culture plates.

1.8 Cell treatment and cell transfection

After the Leydig cells reached a density of 70% in the 6-well and 12-well plates, LDL (Yuanye Bio-Technology Co., Ltd, Shanghai, China; 100 µg/mL) and ox-LDL (Yuanye Bio-Technology Co., Ltd, Shanghai, China; 100 µg/mL) was added to the cell culture, and equal volume of buffer solution provided by the reagent manufacturer was added as control. Lipofectamine 3000 was mixed into the cell culture used for cell transfection, which was carried out according to the manufacturer’s instructions. A set of mouse Klk1b21/24/27-specific siRNA (si-Klk1bs) and nonspecific siRNA (NC) were designed and synthesized by RiboBio (Guangzhou, China). The sequence of si-KLK1B21/24/27 is shown in Supplementary Table 4. The overexpressed plasmid and adenovirus vector of Eid3 and Klk1b21 (Supplementary Table 1, Table 2, Table 3) were purchased from Applied Biological Materials (Richmond, BC, Canada), No-load plasmid as control..

1.9 Library preparation for bulk transcriptome sequencing

Leydig cells from the si-Klk1bs and NC groups were collected for RNA extraction using TRIzol reagent (Thermo Fisher, Waltham, MA, USA) following the manufacturer’s instructions. Purified RNA was DNase-treated using the TURBO DNA-free Kit (Thermo Fisher) according to the instructions, and the RNA quality was assessed using TapeStation RNA ScreenTape (Agilent Technologies, Santa Clara, CA, USA). Eight hundred nanograms of DNA-depleted RNA were used for RNA-Seq library preparation using the TruSeq Stranded Total RNA Library Kit with Ribo-Zero Gold for removal of cytoplasmic and mitochondrial ribosomal RNA according to the manufacturer’s instructions. The libraries were then sequenced on an Illumina HiSeq 4000 system using a paired-end 150 bp run.

1.10 Real-time polymerase chain reaction assays

RNA was isolated and purified from testis cells or Leydig cells using an RNA Extraction Kit (Takara Bio, Tokyo, Japan) following the manufacturer’s instructions. Next, approximately 600–800 ng of total RNA was reverse-transcribed to complementary DNA using a PrimeScript RT Reagent Kit (Takara Bio) with gDNA Eraser. The expression levels of the genes were detected by a real-time fluorescence quantitative PCR instrument (Mx3005P; Agilent) using the SYBR Premix Ex Taq II kit (Takara Bio). The procedure parameters were as follows: 95°C for 10 min followed by 40 cycles of 95°C for 20 s, 60°C for 30 s, and 72°C for 20 s(2). The results were analyzed using the 2^-△△Ct^ method with Actb as the reference gene. All primers were synthesized by Comate Biotechnology Co., Ltd (Changchun, China). The sequences are listed in Table S2.

1.11 Western blot analysis and quantification

Leydig cells and testis tissue were lysed in radioimmunoprecipitation assay buffer (Sigma) containing protease/phosphatase inhibitor (Solarbio Life Sciences, Beijing, China) for protein extraction. The total protein content was detected using a BCA Protein Assay Kit (Beyotime Biotechnology, Jiangsu, China). Equal amounts of protein (approximately 20 µg) were subjected to electrophoresis on 12% Criterion polyacrylamide gels (Shanghai Yeasen Biotech, Shanghai, China) under reducing conditions and transferred to polyvinylidene difluoride membranes (Merck KGaA, Darmstadt, Germany). The membranes were then blocked for 1 h with 5% bovine serum albumin (Solarbio) in Tris-buffered saline (pH 7.4). The membranes were washed three times for 8 min each time in Tris-buffered saline containing 0.05% Tween 20 and probed overnight at 4°C with the primary rabbit anti-human antibodies for Actb, Eid3, Cyp11a1, and Hsd3b1 or the primary goat anti-human antibody for Klk1b27 at 4°C overnight. Following incubation with the primary antibody, the membranes were washed three times for 8 min each time in Tween 20 and incubated for 1 h with horseradish peroxidase-conjugated goat anti-rabbit IgG (1:8,000; Bioworld, Irvine, CA, USA) or donkey anti-goat IgG (1:8,000; Bioworld). After the excess secondary antibodies on the polyvinylidene difluoride membranes were removed, the immunoreactions were detected using an enhanced chemiluminescence detection kit (Beyotime Biotechnology). Images were obtained with a Tanon-5200 fully automated digital gel imaging analysis system (Shanghai Tanon Technology, Shanghai, China).

1.12 Enzyme-linked immunosorbent assays

Cell culture medium and mice serum were collected and frozen at -20°C until analysis of testosterone, LDL, and ox-LDL using ELISA kits according to the instructions. Determination of LDL and ox-LDL in testicular tissue was performed as previously described (3). Briefly, 0.02 g of mouse testis tissue was homogenized with 200 µL of ice-cold radioimmunoprecipitation assay buffer and centrifuged at 12,000 rpm for 10 min at 4°C. The supernatant was collected into a new EP tube. Next, the content of LDL and ox-LDL in the supernatant was determined according to the kit instructions. The results were quantified at a wavelength of 450 nm using an 800 Universal Microplate Reader (BioTek, Winooski, VT, USA).

1.13 Intracellular mitochondrial membrane potential (MMP) assays

The MMP of Leydig cells was measured using a JC-1 (KeyGEN, Nanjing, China) dual-emission membrane potential-sensitive probe that exists as a green fluorescent monomer at a low MMP and forms aggregates with red/orange fluorescence at a high MMP. For imaging, Leydig cells were cultured in 12-well plates, washed three times with PBS, and JC-1 was used as per the instructions. The change in fluorescence at 488/530 nm (green) and 549/595 nm (red) was monitored by high-content screening, and the red fluorescence intensity was determined. The MMP of Leydig cells was measured by JC-1 fluorescent staining were cultured in 6-well plate. Briefly, the Leydig cells were collected into an EP tube and suspended in 1 mL of PBS containing JC-1 10 mg/mL and incubated for 15 min at 37°C in the dark. The cells were then washed three times with 1 mL of PBS and analyzed by flow cytometry on a FACSCalibur cytometer (Becton-Dickinson, San Jose, CA, USA). At least 10,000 Leydig cells were examined, and the data were analyzed using Cellquest Pro software (Becton-Dickinson).

1.14 Adenovirus vector

Adenovirus vectors containing GFP downstream of a CMV promoter or a Cyp17a1 promoter were obtained from Applied Biological Materials. Adenovirus vectors carrying CMV promoters were used to knock down the Klk1bs in the testis. Adenovirus vectors carrying Cyp17a1 promoters were used to specifically overexpress Eid3 or Klk1b21 in Leydig cells. Virus with no-load plasmid as control. Viral particles were supplied at a titer of 1 × 10^10^ PFU/mL.

1.15 Testicular injections

A customized needle was made to inject the adenovirus. Briefly, the needle was removed from a 1-mL insulin syringe and fitted to the head of a white gun. During interstitial injection of the testis, the mice were anesthetized and disinfected, after which the abdominal cavity was opened, and the testis was carefully removed using eyebrow tweezers. Our injection needle was mounted on a 10-µL pipetting gun to inject 10 µL of adenovirus into the mouse testis with a virus titer of >1 × 10^10^ PFU/mL. After the wound was sutured, the mice were returned to the animal house.

1.16 Immunohistochemistry

Testis sections were pretreated with heat-mediated antigen retrieval in sodium citrate buffer (pH 6.0) for 15–20 min, incubated with antibodies against Dazl, Scp3, Tnp1, or Ddx4, and incubated overnight at 4°C. Immunochemical staining was performed using an IHC kit (MXb, CN) and a DAB kit (MXb, CN) according to the manufacturer’s instructions. Finally, the testis tissue sections were observed under an E100 light microscope (Nikon, Tokyo, Japan), and images were obtained using a 600D Photo Imaging System (Canon Inc., Tokyo, Japan).

1.17 Glucose tolerance test

In the mouse glucose tolerance test, the dose of glucose was 2 g/kg. A 20% glucose solution was prepared with normal saline. Before the experiment, the mice were fasted for 16 h but had a normal water intake. After measurement of the blood glucose level, a glucose solution (based on body weight) was injected intraperitoneally. Blood glucose was measured and recorded at 15, 30, 60, 90, and 120 min after the injection.

1.18 Insulin tolerance test

An insulin dose of 0.5 IU/kg was used for the insulin tolerance test. An insulin solution at a concentration of 0.05 IU/mL was prepared with normal saline. Before the experiment, the mice were fasted for 4 h with access to normal amounts of water. After the blood glucose level was measured, the mice received an intraperitoneal injection of insulin solution according to body weight. Blood glucose was measured and recorded at 15, 30, 45, and 60 min after the injection.

1.19 Sperm count

After the mice were euthanized, the intact epididymis was harvested from both sides and placed in a 35-mm plate filled with 1 mL of modified Tyrode’s solution preheated to 37°C. The tissues were cut longitudinally with ophthalmic scissors to release the sperm into the solution. The cells were shaken gently and evenly and cultured in a cell incubator at 37°C with 5% carbon dioxide for 30 min, after which the epididymal tissue was removed. The sperm count was quantified using a hemocytometer under a light microscope.

1.20 Statistical analysis

All data are expressed as the mean ± standard error of the mean. The significance of between-group differences was examined using the unpaired *t*-test and one-way analysis of variance. The statistical analysis was performed using SPSS version 19.0 (IBM Corp., Armonk, NY, USA). P-values of <0.05 and <0.01 were considered statistically significant.

**2 Result**

**Cell type annotation of adult mouse testis cells**

The results of unsupervised clustering showed that the testicular cells of the Control and HFD mice were well distributed throughout the whole cell cluster, and the proportion of the specific cell clusters was closely related to their biological significance. Dazl, Stra8, and Sohlh2 (4-10) were highly expressed in cluster 0 (Fig. 1E-F). Accordingly, this cell cluster was identified as spermatogonia (SPG). Piwil1 and Tex19.2, which are involved in the repression and mobilization of retrotransposons during spermatogenesis (11) and are related to male infertility (12), were highly expressed in cluster 1. Therefore, cluster 1 was defined as the primary spermatocyte cluster (ESC). Clgn, Ccna1, Meioc, Pou5f2, Cdk1, Sycel1 and Syce3 (12-19) were specifically and highly expressed in clusters 2, 3, 4, 5 and 6, respectively. The high expression of these genes indicates that clusters 2-6 were secondary spermatocyte clusters (cluster 2: LSC-1s; cluster 3: LSC-2s; cluster 4: LSC-3s; cluster 5: LSC-4s; and cluster 6: LSC-5s). Clusters 7 and 8 expressed high levels of Spag6 and Tex29, which are markers of round sperm cells and early-stage sperm cells, respectively (13-15). Therefore, clusters 7 and 8 can be defined as round sperm clusters (RS-1 and RS-2). Clusters 9, 10, and 11 specifically expressed the sperm elongation-related genes Fam71b, Prm1, Cdkn1c (p57kip2) and Car2, which are expressed in prolonging cells(6, 16-18). Based on these results, clusters 9, 10, and 11 were defined as different stages of elongating sperm clusters (ES-1, ES-2, ES-3). Sperm cells express high levels of Azin229, and Azin2 was highly expressed in cluster 12, indicating that this cluster belongs to the terminal stage of sperm development, so it was defined as a concentrated sperm cluster (CS). Furthermore, cluster 13 specifically expressed high levels of Cyp17a1, Star and Hsd*, which are related to sterol synthesis and are both marker genes for sex hormone synthesis (20, 21). Based on this result, cluster 13 was testicular Leydig cells (LC). Cst9and Cldn11 (22, 23) are specifically highly expressed in testicular Sertoli cells. Therefore, cluster 14 was defined as Sertoli cells; cluster 15 mainly expressed the Zfp389 and Elfn2 genes that are related to stimulation and shared genes with other clusters. Therefore, a significant marker gene could not be identified for this cluster. Based on this result, cluster 15 was defined as testicular dendritic cell-like (DC-like). According to the gene expression dynamics of these marker genes, we identified 16 clusters of cells in adult mouse testes, including male germ cells ranging from spermatogonia to spermatids and four types of testicular somatic cells (Fig. 1G).

**3 References**

1. Matzkin, M. E., Yamashita, S., and Ascoli, M. (2013) The ERK1/2 pathway regulates testosterone synthesis by coordinately regulating the expression of steroidogenic genes in Leydig cells. *Molecular and cellular endocrinology* **370**, 130-137

2. Yuan, C., Li, Z., Zhao, Y., Wang, X., Chen, L., Zhao, Z., Cao, M., Chen, T., Iqbal, T., Zhang, B., Fan, W., Wei, Y., Li, C., and Zhou, X. (2021) Follicular fluid exosomes: Important modulator in proliferation and steroid synthesis of porcine granulosa cells. *FASEB journal : official publication of the Federation of American Societies for Experimental Biology* **35**, e21610

3. Jing, J., Ding, N., Wang, D., Ge, X., Ma, J., Ma, R., Huang, X., Jueraitetibaike, K., Liang, K., Wang, S., Cao, S., Zhao, A. Z., and Yao, B. (2020) Oxidized-LDL inhibits testosterone biosynthesis by affecting mitochondrial function and the p38 MAPK/COX-2 signaling pathway in Leydig cells. *Cell death & disease* **11**, 626

4. González, C. R., Moverer, L., Calandra, R. S., González-Calvar, S. I., and Vitullo, A. D. (2018) Age-related and photoperiodic variation of the DAZ gene family in the testis of the Syrian hamster (Mesocricetus auratus). *Zygote (Cambridge, England)* **26**, 127-134

5. Mikedis, M. M., Fan, Y., Nicholls, P. K., Endo, T., Jackson, E. K., Cobb, S. A., de Rooij, D. G., and Page, D. C. (2020) DAZL mediates a broad translational program regulating expansion and differentiation of spermatogonial progenitors. *eLife* **9**

6. Liu, W., Wang, F., Xu, Q., Shi, J., Zhang, X., Lu, X., Zhao, Z. A., Gao, Z., Ma, H., Duan, E., Gao, F., Gao, S., Yi, Z., and Li, L. (2017) BCAS2 is involved in alternative mRNA splicing in spermatogonia and the transition to meiosis. *Nat Commun* **8**, 14182

7. Niu, C., Guo, J., Shen, X., Ma, S., Xia, M., Xia, J., and Zheng, Y. (2020) Meiotic gatekeeper STRA8 regulates cell cycle by interacting with SETD8 during spermatogenesis. *Journal of cellular and molecular medicine* **24**, 4194-4211

8. Shen, X., Niu, C., Guo, J., Xia, M., Xia, J., Hu, Y., and Zheng, Y. (2018) Stra8 may inhibit apoptosis during mouse spermatogenesis via the AKT signaling pathway. *International journal of molecular medicine* **42**, 2819-2830

9. Toyoda, S., Miyazaki, T., Miyazaki, S., Yoshimura, T., Yamamoto, M., Tashiro, F., Yamato, E., and Miyazaki, J. (2009) Sohlh2 affects differentiation of KIT positive oocytes and spermatogonia. *Developmental biology* **325**, 238-248

10. Barrios, F., Filipponi, D., Campolo, F., Gori, M., Bramucci, F., Pellegrini, M., Ottolenghi, S., Rossi, P., Jannini, E. A., and Dolci, S. (2012) SOHLH1 and SOHLH2 control Kit expression during postnatal male germ cell development. *Journal of cell science* **125**, 1455-1464

11. Tarabay, Y., Achour, M., Teletin, M., Ye, T., Teissandier, A., Mark, M., Bourc'his, D., and Viville, S. (2017) Tex19 paralogs are new members of the piRNA pathway controlling retrotransposon suppression. *Journal of cell science* **130**, 1463-1474

12. Soh, Y. Q. S., Mikedis, M. M., Kojima, M., Godfrey, A. K., de Rooij, D. G., and Page, D. C. (2017) Meioc maintains an extended meiotic prophase I in mice. *PLoS genetics* **13**, e1006704

13. Siep, M., Sleddens-Linkels, E., Mulders, S., van Eenennaam, H., Wassenaar, E., Van Cappellen, W. A., Hoogerbrugge, J., Grootegoed, J. A., and Baarends, W. M. (2004) Basic helix-loop-helix transcription factor Tcfl5 interacts with the Calmegin gene promoter in mouse spermatogenesis. *Nucleic acids research* **32**, 6425-6436

14. Lele, K. M., and Wolgemuth, D. J. (2004) Distinct regions of the mouse cyclin A1 gene, Ccna1, confer male germ-cell specific expression and enhancer function. *Biol Reprod* **71**, 1340-1347

15. Zini, A., Mielnik, A., and Schlegel, P. N. (1996) POU-domain gene expression during spermatogenesis. *World journal of urology* **14**, 274-277

16. Clement, T. M., Inselman, A. L., Goulding, E. H., Willis, W. D., and Eddy, E. M. (2015) Disrupting Cyclin Dependent Kinase 1 in Spermatocytes Causes Late Meiotic Arrest and Infertility in Mice. *Biol Reprod* **93**, 137

17. Okazaki, R., Yamazoe, K., and Inoue, Y. H. (2020) Nuclear Export of Cyclin B Mediated by the Nup62 Complex Is Required for Meiotic Initiation in Drosophila Males. *Cells* **9**

18. Sciurano, R. B., Pigozzi, M. I., and Benavente, R. (2019) Disassembly of the synaptonemal complex in chicken oocytes analyzed by super-resolution microscopy. *Chromosoma* **128**, 443-451

19. Hernández-Hernández, A., Masich, S., Fukuda, T., Kouznetsova, A., Sandin, S., Daneholt, B., and Höög, C. (2016) The central element of the synaptonemal complex in mice is organized as a bilayered junction structure. *Journal of cell science* **129**, 2239-2249

20. Liu, Y., Zhang, L., Li, W., Huang, Q., Yuan, S., Li, Y., Liu, J., Zhang, S., Pin, G., Song, S., Ray, P. F., Arnoult, C., Cho, C., Garcia-Reyes, B., Knippschild, U., Strauss, J. F., and Zhang, Z. (2019) The sperm-associated antigen 6 interactome and its role in spermatogenesis. *Reproduction (Cambridge, England)* **158**, 181-197

21. Zhang, H. W., Zhang, L., Chen, Y. M., Shen, X., Wang, H. Q., Liu, Y. H., and Zhang, Z. B. (2019) [The role of SPAG6/SPINK2 protein complex in the formation of sperm acrosome in mice]. *Zhonghua nan ke xue = National journal of andrology* **25**, 202-208

22. Qu, N., Nagahori, K., Kuramasu, M., Ogawa, Y., Suyama, K., Hayashi, S., Sakabe, K., and Itoh, M. (2020) Effect of Gosha-Jinki-Gan on Levels of Specific mRNA Transcripts in Mouse Testes after Busulfan Treatment. *Biomedicines* **8**

23. Ernst, C., Eling, N., Martinez-Jimenez, C. P., Marioni, J. C., and Odom, D. T. (2019) Staged developmental mapping and X chromosome transcriptional dynamics during mouse spermatogenesis. *Nat Commun* **10**, 1251
