# Supplementary figures and images for: Increased oxidized low‐density lipoprotein in mice exposed to a high‐fat diet impaired spermatogenesis by inhibiting testosterone synthesis via the Klk1bs/Eid3 pathway
Source: Clin Transl Med. 2024 Mar 3;14(3):e1603. doi: 10.1002/ctm2.1603 (PMC10909978; doi:10.1002/ctm2.1603)

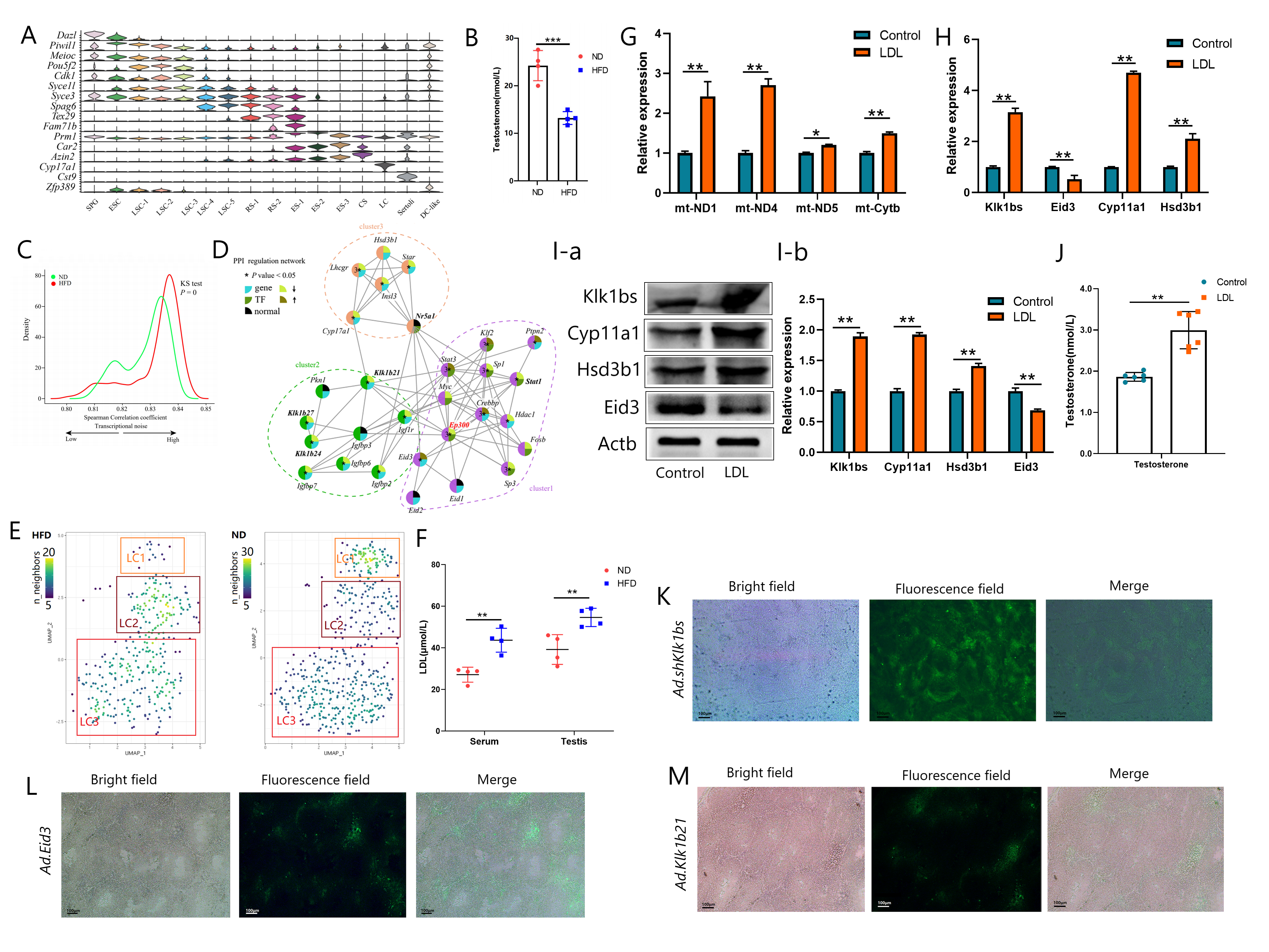

Supplement: Supplementary file 2 — Figure S1: High‐fat diet impaired spermatogenesis by inhibiting testosterone synthesis. [file CTM2-14-e1603-s002.tiff]
