# Supplementary material for: Increased oxidized low‐density lipoprotein in mice exposed to a high‐fat diet impaired spermatogenesis by inhibiting testosterone synthesis via the Klk1bs/Eid3 pathway
Source: Clin Transl Med. 2024 Mar 3;14(3):e1603. doi: 10.1002/ctm2.1603 (PMC10909978; doi:10.1002/ctm2.1603)
Supplement: Supplementary file 3 — Supporting Information [file CTM2-14-e1603-s001.docx]

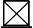
**Supplementary Table 1**：Custom shRNA pAdeno Vector


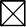


| Vector  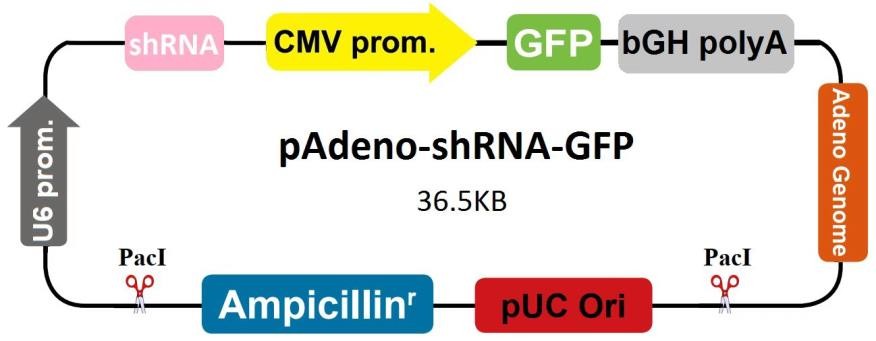 | | | | | | | | | | | |
| --- | --- | --- | --- | --- | --- | --- | --- | --- | --- | --- | --- |
|  | Name: | pAdeno-shRNA-GFP | | | | | | | | | |
|  | Size: | 36.5kb | | | Cloning Site(s): | | | N/A | | | |
|  | Promoter: | | | | Reporter: | | | Selection Marker in Bacteria: | | | |
|  | CMV EF1α | | PGK  UbC | | GFP RFP | | | Kanamycin 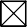Ampicillin | | Spectinomycin Chloramphenicol | |
|  | 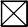 Other: U6 for shRNA | | | | Other: | | | Other: | | | |
|  | Tag: | | |  | | | | Selection Marker in Mammalian Cells: | | | |
|  | N-term His | | | HA | | | | Puromycin | | | Neomycin |
|  | C-term His | | | 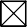 Other: N/A | | | | 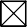 Other: N/A | | | |
|  | Competent Cells: | | | | | | | | | | |
|  | DH5α | | | | | | EPI400 | | | | |
|  | TOP10 | | | | | | Other: | | | | |
| Insert | | | | | | | | | | | |
| Name: | | Custom shRNA: CTGACTACAACATGAGCCTTTCAAGAGAAGGCTCATGTTGTAGT CAG | | | | | | | | | |
| Size: | | 47bp | | | | Cloning Site(s): | | | N/A | | |

**Supplementary Table 2**：Cyp17a1-promoter-Eid3-CBH-GFP pAdeno Vector (Mouse)


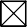


| Vector  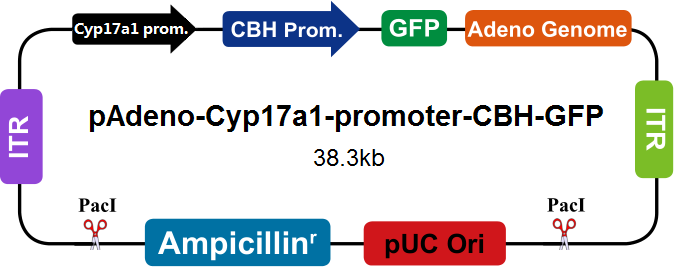 | | | | | | | | |
| --- | --- | --- | --- | --- | --- | --- | --- | --- |
|  | Name: | pAdeno-Cyp17a1-promoter-CBH-GFP | | | | | | |
|  | Size: | 38.3kb | | | Cloning Site(s): | N/A | | |
|  | Promoter: | | | | Reporter: | Selection Marker in Bacteria: | | |
|  | CMV EF1α | | PGK  UbC | | GFP RFP | Kanamycin 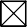Ampicillin | Spectinomycin Chloramphenicol | |
|  | 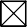 Other: Cyp17a1 promoter | | | | Other: | Other: | | |
|  | Tag: | | |  | | Selection Marker in Mammalian Cells: | | |
|  | N-term His | | | HA | | Puromycin | | Neomycin |
|  | C-term His | | | 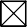 Other: N/A | | 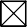 Other: N/A | | |

| Insert | | | |
| --- | --- | --- | --- |
| Name: | Orange: Kozak Red: Stop codon | | |
| Eid3 | GCCACCATGTCTAAAGAAAAATGTTCCCTTACTGGAGGCAAAGAGAAAAGAGGGGAGC | | |
|  | TGGTCCGGAGTCTGGCATGGCAGCACCTGGTGAAACAGGAGGAGGAAGAGGCAGTG | | |
|  | AAGAAGGAAGAAAAGGAGGAAGGGGAAGATGAGGAGGAGGAAGGCTCAGACAGTAG | | |
|  | CTCCGATGACCCGAACCCCGAGCCCCCCTGCATGCACCCAGACCTCCTGGAGCTGGT | | |
|  | GGTGGATCGAGAGAAGTGCCGCAAAATCCGCAGGCAGTACCGGCAGCTCATCTACAC | | |
|  | CGTGCAGCAGAACCGCGAGGACATTGTGAACACGGCCAGCGACACGCTGAGTGAGG | | |
|  | CCCTGGAGGAAGCCAACGTGCTGTTTGACGGAGTGAGCAGAACCAGAGAGGCAGCCC | | |
|  | TTGATGCCCAGTTCCTGGTTTTGGCCTCTGATCTGGGTAAAGAGAAGGCGAAGCAGCT | | |
|  | AAACACCGATATGAACTTTTTTAATCCCATTGCCTTCTGCGATTTGCTGCTGTTGTTTGT | | |
|  | GGGCTTCAATTGGGTAGAAGAGGAGTGTAAGGAATTTAGCGACTGCGATGATAGCATA | | |
|  | GTTCTTTCCTTTTGGGGCATGTTGCACGAGGAAGCAACCTCCTGGATGCTGCAAGCCG | | |
|  | AAACGTTCCACTTCATTTTTGGGTCATTTAAGGCAGAACGTTCTGCACGAAAGCCCCGG | | |
|  | CTTGGATGTCACAAAAGAGCTTGCAAAATGGAAGGAAGTGGAGATATGCCTACAAAGT | | |
|  | TGAGGAGGCTGGATGTGCATGCTAATCAGGAGACGACAGAAAAAGAAGTTGAGAGAAT | | |
|  | CTTGGGATTGCTGCAAACCTACTTTCAAAAGTACCCCGATACTCCAGTGTCATACTTTG | | |
|  | AGTTTGTGATTGATCCAAACTCATTCTCTCGCACTGTGGAGAATATCTTCTATGTGTCTT | | |
|  | TTATTATTAGGGATGGCTTTGCAAGAATAAGGCTTGACCAAGACAGGCTGCCAATTCTA | | |
|  | GAGCCAACTAATGTGAGCCAGGTGGATGATGAAAGTGATTCCTATTCGTACTGCAGGA | | |
|  | AACAAGGCGTTATATCTTTGAGTTTACAGGACTGGAAAAATATTGTCTCCACTTTTGAAA | | |
|  | TTTCAGAGGCTATGATCAAAAACTCATATTAA | | |
| Size: | 1134bp | Cloning Site(s): | N/A |


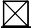
**Supplementary Table 3**：Cyp17a1 promoter-Klk1b21-CBH-GFP pAdeno Vector (Mouse)


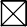


| Vector  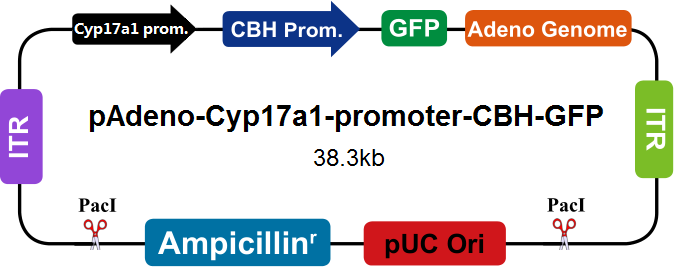 | | | | | | | | | | | | |
| --- | --- | --- | --- | --- | --- | --- | --- | --- | --- | --- | --- | --- |
|  | Name: | | pAdeno-Cyp17a1-promoter-CBH-GFP | | | | | | | | | |
|  | Size: | | 38.3kb | | | Cloning Site(s): | | | N/A | | | |
|  | Promoter: | | | | | Reporter: | | | Selection Marker in Bacteria: | | | |
|  | CMV EF1α | | | PGK  UbC | | GFP RFP | | | Kanamycin 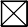Ampicillin | | Spectinomycin Chloramphenicol | |
|  | 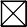 Other: Cyp17a1 promoter | | | | | Other: | | | Other: | | | |
|  | Tag: | | | |  | | | | Selection Marker in Mammalian Cells: | | | |
|  | N-term His | | | | HA | | | | Puromycin | | | Neomycin |
|  | C-term His | | | | 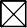 Other: N/A | | | | 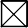 Other: N/A | | | |
|  | Competent Cells: | | | | | | | | | | | |
|  | DH5α | | | | | | | EPI400 | | | | |
|  | TOP10 | | | | | | | Other: | | | | |
| Insert | | | | | | | | | | | | |
| Name:  klk1b21 | | Orange: Kozak Red: Stop codon GCCACCATGAGGTTCCTGATCCTGTTCCTTGCCCTGTCCCTAGGAGAGATTGATGCTG CACCTCCTGTCCAGTCTCGAATTGTTGGAGGATTTAACTGTGAGAAGAATTCTCAACCC TGGCATGTGGCTGTGTTCCGCTACAACAAATATATATGCGGGGGAGTCCTGTTGAACC CCAACTGGGTTCTCACAGCTGCCCACTGCTATGGCAACCAGTATAATGTTTGGCTGGG CAAAAACAAGCTATTCCAACATGAATCCTCTGCTCAGCACCGATTGGTCAGCAAAAGCT TCCCTCACCCTGACTACAACATGAGCCTCATGAATGACCACACCCCACATCCTGAGGA TGACTACAGCAATGACCTGATGTTACTACGCCTCAGCAAGCCTGCTGACATCACAGAT GCTGTGAAGCCCATCGACCTGCCCACTGAGGAGCCCAAGCTGGGGAGCACATGCCTA GCCTCAGGCTGGGGCAGCATTACACCCACGAAATGGCAAATCCCAAATGATCTCCAGT GTGGGTTCATCAAGCCCCTGCCTAATGAAAATTGTGCCAAAGCCTACATACATAAAGTC ACAGATGTCATGCTGTGTGCAGGAGAGATGGGTGGAGGCAAAGACACTTGTGCGGGT GACTCAGGAGGCCCACTGATCTGTGATGGTGTTCTACAAGGTATCACATCATGGGGCT CTATCCCATGCGCTAAACCCAATGCGCCGGCCATCTACACCAAACTTATTAAGTTTACC TCCTGGATAAAAGACACTATGGCCAAAAACCCCTGA | | | | | | | | | | |
| Size: | | 792bp | | | | | Cloning Site(s): | | | N/A | | |

**Supplementary Table 44：** siRNA sequences

| Accession | siRNA name | siRNA sequence |
| --- | --- | --- |
| siG2004140217205222 | si-m-Klk1b24/Klk1b21/Klk1b27_001 | GACATCACAGATGCTGTGA |
| siG2004140217206314 | si-m-Klk1b24/Klk1b21/Klk1b27_002 | CCCACTGATCTGTGATGGT |
| siG2004140217207406 | si-m-Klk1b24/Klk1b21/Klk1b27_003 | CTGACTACAACATGAGCCT |

**Supplementary Table 5：** Sequences of primers used for real-time quantitative polymerase chain reaction

| Gene symbol | Forward sequence | Reverse sequence |
| --- | --- | --- |
| *Actb* | F‐5′GTGACGTTGACATCCGTAAAGA3′ | R‐5′GCCGGACTCATCGTACTCC3′ |
| *Klk1b21* | F‐5′GTCTCCCGAGGATCTTCAACA3′ | R-5′GGAGGTGCAGCATCAATCTCT3′ |
| *Klk1b24* | F-5′TGGCAAAAGCCAAATGACCTT3′ | R-5′ATTGGGTTTACCACATGGGAC3′ |
| *Klk1b27* | F-5′GCCTCCTGTCCAGTCATGAATAA3′ | R-5′GGCTGGTGTCATTGCCATA3′ |
| *Eid3* | F-5′TTGAGAGAATCTTGGGATTGCTG3′ | R‐5′CACAGTGCGAGAGAATGAGTTT3′ |
| *Cyp11a1* | F-5′AGTATTATCAGAGGCCCATTGG3′ | R‐5′AACATCTGGTAGACAGCATTGA3′ |
| *Hsd3b1* | F-5′AAAGGTACCCAGAACCTATTGG3′ | R‐5′CTGTATGGGTATGGATCAGACC3′ |
| *Cxcl1* | F-5′ACTGCACCCAAACCGAAGTC3′ | R‐5′TGGGGACACCTTTTAGCATCTT3′ |
| *Klk1b21/24/27* | F‐5′GGCAGCATTACACCCACGA3′ | R-5′TGCCTCCACCCATCTCTCCT3′ |
| *mt-ND1* | F‐5′CTAATCGCCATAGCCTTCCT3′ | R-5′AGTTGTTAAAGGGCGTATTGGTT3′ |
| *mt-ND4* | F‐5′GGATCCACAGCCGTACTATAAT3′ | R‐5′TGAAGGGGGTAGAGCTAGATTA3′ |
| *mt-ND5* | F‐5′TTCTCCAACAACAACGACAATC3′ | R‐5′TCCGAGGCAAAGTATAGTTGTT3′ |
| *mt-Cytb* | F‐5′ACGCAAACGGAGCCTCAATA3′ | R‐5′CCTCATGGAAGGACGTAGCC3′ |
